# Comparative Analysis of Fatty Acid Desaturases in Cyanobacterial Genomes

**DOI:** 10.1155/2008/284508

**Published:** 2008-12-02

**Authors:** Xiaoyuan Chi, Qingli Yang, Fangqing Zhao, Song Qin, Yu Yang, Junjun Shen, Hanzhi Lin

**Affiliations:** ^1^Key Laboratory of Experimental Marine Biology, Institute of Oceanology, Chinese Academy of Sciences, Qingdao 266071, China; ^2^Graduate University, Chinese Academy of Sciences, Beijing 100039, China; ^3^Shandong Peanut Research Institute, Qingdao 266100, China

## Abstract

Fatty acid desaturases are enzymes that introduce double bonds into the hydrocarbon chains of fatty acids. The fatty acid desaturases from 37 cyanobacterial genomes were identified and classified based upon their conserved histidine-rich motifs and phylogenetic analysis, which help to determine the amounts and distributions of desaturases in cyanobacterial species. The filamentous or N_2_-fixing cyanobacteria usually possess more types of fatty acid desaturases than that of unicellular species. The pathway of acyl-lipid desaturation for unicellular marine cyanobacteria *Synechococcus* and *Prochlorococcus* differs from that of other cyanobacteria, indicating different phylogenetic histories of the two genera from other cyanobacteria isolated from freshwater, soil, or symbiont. Strain *Gloeobacter violaceus* PCC 7421 was isolated from calcareous rock and lacks thylakoid membranes. The types and amounts of desaturases of this strain are distinct to those of other cyanobacteria, reflecting the earliest divergence of it from the cyanobacterial line. Three thermophilic unicellular strains, *Thermosynechococcus elongatus* BP-1 and two *Synechococcus* Yellowstone species, lack highly unsaturated fatty acids in lipids and contain only one Δ9 desaturase in contrast with mesophilic strains, which is probably due to their thermic habitats. Thus, the amounts and types of fatty acid desaturases are various among different cyanobacterial species, which may result from the adaption to environments in evolution.

## 1. Introduction

In living organisms, the regulation of membrane fluidity is necessary for the proper function of biological membranes, which is important in the tolerance and acclimatization to environmental stresses such as heat, cold, desiccation, salinity, nitrogen starvation, photooxidation, anaerobiosis, and osmosis, and so forth. Unsaturated fatty acids are essential constituents of polar glycerolipids in biological membranes and the unsaturation level of membrane lipids is important in controlling the fluidity of membranes [1]. Fatty acid desaturases are enzymes that introduce double bonds into the hydrocarbon chains of fatty acids to produce unsaturated and polyunsaturated fatty acids [2], thus these enzymes play an important role during the process of environmental adaptation.

Cyanobacteria, prokaryotes capable of carrying out a plant-like oxygenic photosynthesis, represent one of the oldest known bacterial lineages, with fossil evidence suggesting an appearance around 3–3.5 billion years ago [3]. Cyanobacteria comprise over 1600 species with various morphologies and species-specific characteristics such as cell movement, cell differentiation, and nitrogen fixation [4]. Extant cyanobacteria can be found in virtually all ecosystem habitats on Earth, ranging from the freshwater lakes and rivers through to the oceans, and also in hot springs and deserts, ranging from the hottest to the cold dry valleys of Antarctica [3].

Polyunsaturated membrane lipids play important roles in the growth, respiration, and photosynthesis of cyanobacteria. It is well documented that the content of polyunsaturated fatty acids in membrane lipids of cyanobacteria can be altered by changing the temperature [5–7]. The mechanism that regulates the fatty acid desaturation of membrane lipids in response to temperature has been demonstrated to be the result of the up- or downregulation of the expression of the desaturase genes [8]. Furthermore, it has been demonstrated that the position of double bonds in fatty acids is more influential on the fluidity of membrane lipids than the number of double bonds in fatty acids [9]. It is also found that the temperature of the phase transition dramatically decreased when the first and second double bonds are introduced into fatty acids, whereas the introduction of the third and fourth double bonds do not further lower the temperature of phase transition of membrane lipids [10].

Exposure of cyanobacteria to high PAR (photosynthetically active radiation) or UV radiation leads to photoinhibition of photosynthesis, thereby limiting the efficient fixation of light energy [11, 12]. In *Synechocystis* sp. PCC 6803, the replacement of all polyunsaturated fatty acids by a monounsaturated fatty acid suppressed the growth of the cells at low temperature, and it decreased the tolerance of the cells to photoinhibition of photosynthesis at low temperature by suppressing recovery of the photosystem II protein complex from photoinhibitory damage. However, the replacement of tri- and tetraunsaturated fatty acids by a diunsaturated fatty acid did not have such effects. These findings indicate that polyunsaturated fatty acids are important in protecting the photosynthetic machinery from photoinhibition at low temperatures [13]. Transformation of the cyanobacterium *Synechococcus* sp. PCC 7942 with the *desA* gene for a Δ12 desaturase has been reported to increase the unsaturation of membrane lipids and thereby enhance the tolerance of cyanobacterium to intense light. These findings demonstrate that the ability of membrane lipids to desaturate fatty acids is important for the photosynthetic organisms to be able to tolerate high-light stress by accelerating the synthesis of the D_1_ protein *de novo* [14].

Cyanobacteria have been classified into four groups in terms of the composition of fatty acids, the distribution of fatty acids at the *sn* position of the glycerol moiety, and the position of double bonds in the fatty acids [15]. Strains in Group 1 (e.g., * Prochlorothrix hollandica*, *Synechococcus* sp. PCC 6301, *Synechococcus* sp. PCC 7942, *Synechococcus elongatus*, *Thermosynechococcus elongates*, and *Thermosynechococcus vulcanus*) introduce a double bond only at the Δ9 position of fatty acids at the *sn*-1 or *sn*-2 position of glycerolipids. Strains in Group 2 (e.g., *Anabaena variabilis*, *Anabaena* sp. PCC 7120, * Synechococcus* sp. PCC 7002, *Nostoc punctiforme,* and * Nostoc* sp. SO-36) introduce double bonds at the Δ9, Δ12, and Δ15 (*ω*3) positions of C18 acids at the *sn*-1 position, and at the Δ9 position of C16 acids at the *sn*-2 position. Strains in Group 3 (e.g., *Synechocystis* sp. PCC 6714 and *Spirulina platensis*) can also introduce three double bonds, but these are at the Δ6, Δ9, and Δ12 positions of C18 acids at the *sn*-1 position. Strains in Group 4 (e.g., *Synechocystis* sp. PCC 6803 and *Tolypothrix tenuis*) introduce double bonds at the Δ6, Δ9, Δ12, and Δ15 (*ω*3) positions of C18 acids at the *sn*-1 position. The C16 acids at the *sn*-2 position are not desaturated in Groups 3 and 4.

**Figure 1:**
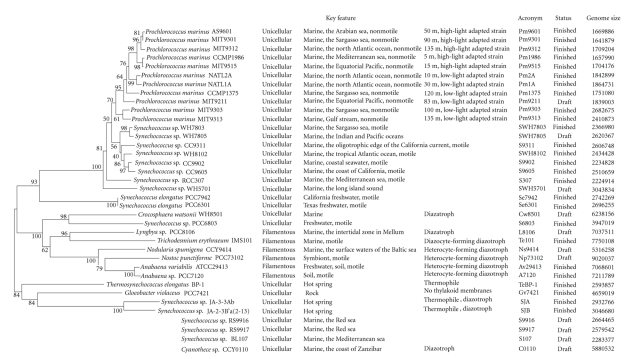
Phylogenetic tree of the sequenced cyanobacterial strains. A Neighbor-joining tree for 33 sequenced cyanobacteria constructed based on 16 S rRNA as was described in Section 2 and about 1300 positions were employed. To maximize the number of sites available for analysis, three partial sequences from *Synechococcus* sp. RS9917 (170 bp), *Synechococcus* sp. RS9916 (865 bp), and *Synechococcus* sp. BL107 (296 bp) were excluded. Moreover, no 16 S rRNA sequence was found in *Cyanothece* sp. CCY0110.

The entire genome sequence of a unicellular cyanobacterium *Synechocystis* sp. strain PCC 6803 was first described in 1996 [16]. To date, 37 cyanobacterial genomes have been sequenced (Figure 1). These genomes are those of the filamentous nitrogen-fixing cyanobacterium *Anabaena* sp. PCC 7120, the thermophilic strain *Thermosynechococcus elongatus* BP-1, the thylakoid-free strain *Gloeobacter violaceus* PCC 7421, the marine cyanobacterium *Synechococcus* sp. strain WH8102, the *Prochlorococcus marinus* strains SS120, MED4, MIT 9313, *Synechococcus* sp. CC9311, and others. These genome-sequencing projects undoubtedly bring a great convenience to obtain a comprehensive dataset of genes involved in unsaturated fatty acid biosynthesis in cyanobacteria. In this work, we identified all the putative fatty acid desaturases using bioinformatic tools and presented a genomic comparison of the fatty acid desaturases from 37 cyanobacterial genomes. The identification of novel desaturases and the reconstruction of the pathways for unsaturated fatty acid biosynthesis in cyanobacteria will guide the experimental analysis and provide clues in study of the relationship between the unsaturation level of membrane lipids and environmental adaptation in higher plants.

## 2. Materials and Methods

### 2.1. Computational Search for Novel Fatty Acid Desaturase Genes

The genomes of 37 cyanobacteria including genera *Synechocystis*, *Synechococcus*, *Prochlorococcus*, *Anabaena*, *Nostoc*, *Trichodesmium,* *Gloeobacter*, *Crocosphaera*, *Cyanothece,* and *Lyngbya* were downloaded from IMG database (http://img.jgi.doe.gov/cgi-bin/pub/main.cgi). The dataset comprised of well-characterized fatty acid desaturases from *Synechocystis* PCC 6803 (NP_442430, NP_441489, NP_441622, NP_441824), *Nostoc* sp. SO-36 (CAF18426), *Synechococcus* sp. PCC 7002 (AAB61353, AAF21445, AAB61352), *Arthrospira platensis* (CAA05166, Q54794, CAA60573), *Synechococcus vulcanus* (AAD00699), *Synechococcus s elongatus* sp. PCC 6301 (YP_172259), *Synechococcus elongatus* sp. PCC 7942 (YP_401578), *Phaeodactylum tricorutum* (AAW70158, AY082393, AAO23565, AY165023), *Chlamydomonas reinhardtii* (AB007640, ABL09485, EDP04777), and *Chlorella vulgaris* (AB075526, AB075527) was used to construct a query protein set. Each protein in this query dataset was used to search the potential novel sequences in 37 cyanobacterial species with whole genome sequences available, by using the BLASTP and TBLASTN programs, with *E*-value < 1*e* − 10. The searches were repeated until no novel sequences were detected at the *e* value threshold used. The putative desaturase genes across 37 genomes were summarized in Table 1. The other amino acid sequences beyond the 37 cyanobacterial species were retrieved from NCBI (http://www.ncbi.nlm.nih.gov/). The accession number of these sequences and the names of corresponding cyanobacteria, eukaryotic algae, higher plants, fungi, and animals were indicated in Table 2.

### 2.2. Multiple Sequence Alignment and Phylogenetic Analysis

Sequence alignments were generated using Clustal W program [17]. The SMART (http://smart.embl-heidelberg.de/) and PFAM (http://pfam.sanger.ac.uk/) databases were used to search the conserved domains of the putative desaturase enzymes. The conserved amino acid residues of different conserved domains were manually identified using the BioEdit sequence editor. The final alignment was further refined after excluding the poorly conserved regions at the protein ends, and consisted of sequences spanning the conserved domains. The neighbor-joining (NJ) and minimum-evolution (ME) methods in MEGA4 [18] were used to construct the phylogenetic tree. To maximize the number of sites available for analysis, two partial sequences from *Synechococcus* sp. WH 7805 (ZP_01124768, 174 aa) and *Nodularia spumigena* CCY9414 (ZP_01629726, 196 aa) were excluded. Bootstrap with 1000 replicates was used to establish the confidence limit of the tree branches.

## 3. Results and Discussions

### 3.1. The Conserved Motifs

Using BlastP and TBlastN programs with the query sequences to search the 37 genomes of cyanobacteria, 193 protein sequences were identified including fatty acid desaturase, fatty acid dehydrogenase, hypothetical protein, *β*-carotene ketolase, *β*-carotene hydroxylase, and hydrocarbon oxygenase. PFAM and SMART domain analyses could not distinguish fatty acid desaturase from fatty acid dehydrogenase, *β*-carotene ketolase, *β*-carotene hydroxylase, or hydrocarbon oxygenase. Moreover, most of the protein sequences which were originally annotated as fatty acid desaturase were not classified into Δ9, Δ12, Δ15, or Δ6 desaturase categories. To facilitate the classification of different types of desaturases, the conserved motifs of different enzymes were identified by multiple sequence alignments with Clustal W.

**Figure 2:**
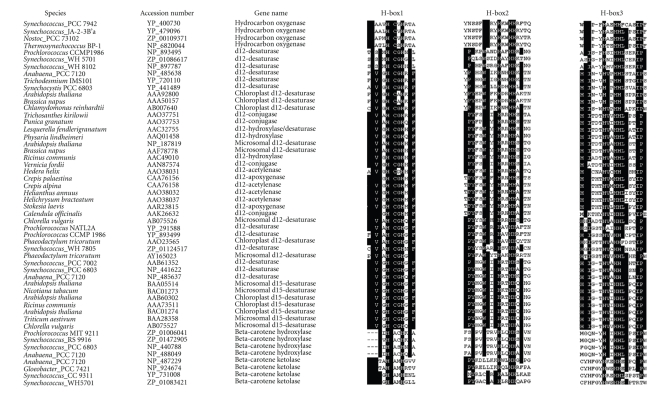
Comparison of the three conserved histidine-rich motifs of proteins from cyanobacteria, eukaryotic algae, and higher plants, including Δ12 fatty acid desaturase, Δ15 fatty acid desaturase, *β*-carotene ketolase, *β*-carotene hydroxylase, hydrocarbon oxygenase, Δ12 fatty acid epoxygenase, Δ12 fatty acid acetylenase, Δ12 fatty acid conjugase, and Δ12 fatty acid hydroxylase. The conserved amino acid residues are in black. “Microsomal” represents the microsome-type desaturases, “Chloroplast” represents the chloroplast-type desaturases.

There were three typical histidine-rich motifs existed in all the proteins similar to proven cyanobacterial fatty acid desaturases (Table 3). Moreover, there were different conserved residues in the same histidine-boxes of different kinds of proteins, suggesting that these proteins might have acquired different functions from a common ancestor during the evolution. According to the different conserved residues of three histidine-motifs and phylogenetic profile, 16 *β*-carotene ketolases, 36 *β*-carotene hydroxylases, and 8 hydrocarbon oxygenases (MocD, a rhizopine oxygenase for the conversion of 3-O-MSI to SI)) were identified from the 37 cyanobacterial genomes (Figures 2, 4, and 5).

### 3.2. Discovery of Candidate Genes for Δ9 Desaturases

To elucidate the phylogenetic relationships among different membrane desaturases, genes from cyanobacteria, eukaryotic algae, higher plants, fungi, invertebrates, and vertebrates were analyzed using neighbor-joining (NJ) and minimum-evolution (ME) methods. Observation of the tree revealed that all the desaturases fell into three distinct subfamilies (Figures 12 and 13): Δ9 desaturase subfamily, Δ12/*ω*3 desaturases subfamily, and the front-end desaturases subfamily.

**Figure 3:**
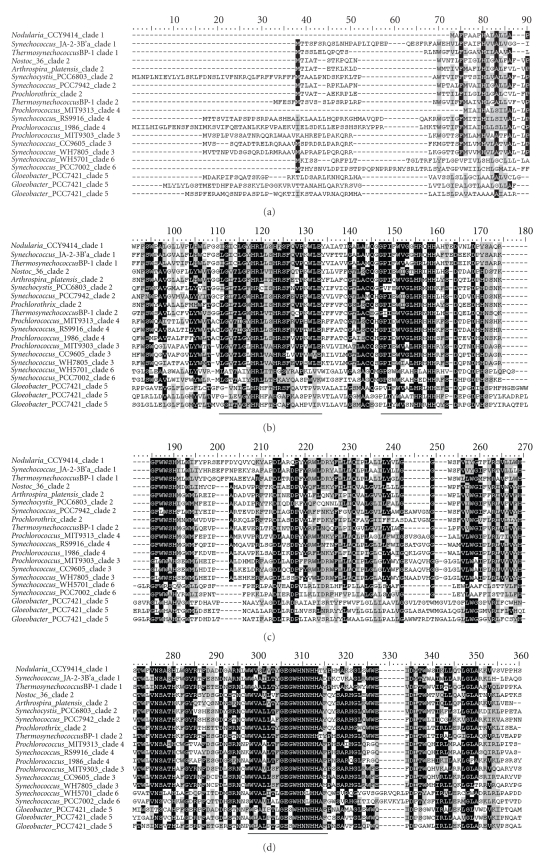
Alignment of the complete deduced amino acid sequences of Δ9-homologous genes. Amino acid residues that are conserved are highlighted in black boxes. The conserved His clusters and their associated conserved domains are underlined. The limits of the domains are indicated by the residue positions, on top of the sequence. The sequences are denoted by their strain names and the clades they belong to.

**Figure 4:**
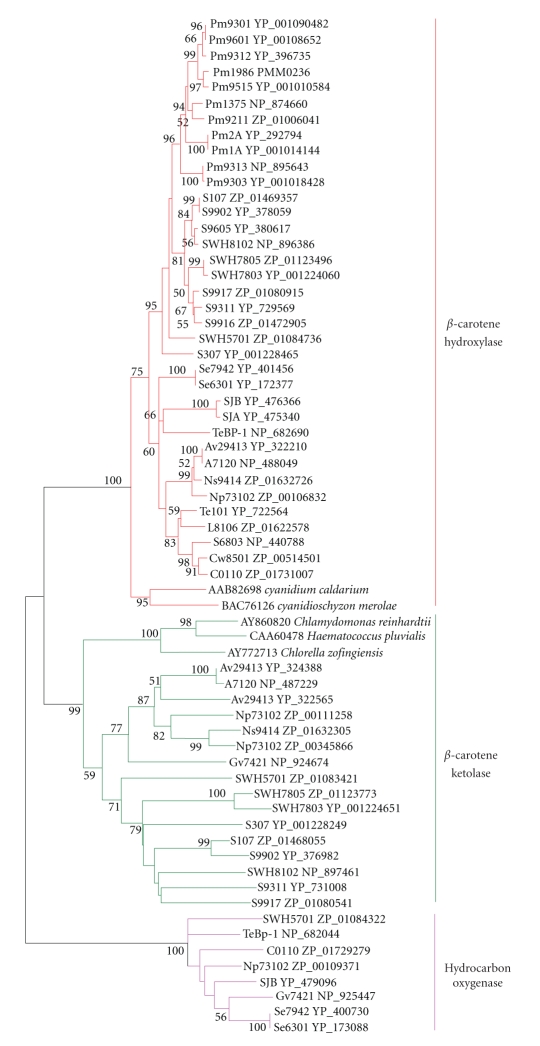
Neighbor-joining tree of *β*-carotene ketolase, *β*-carotene hydroxylase, and hydrocarbon oxygenase homologs of cyanobacteria and eukaryotic algae. About 220 positions spanning the three histidine-boxes were employed. Colored branches indicate different groups of proteins. Red: *β*-carotene hydroxylase, green: *β*-carotene ketolase, magenta: hydrocarbon oxygenase. Sequences from 37 sequenced cyanobacterial genomes are shown by their acronyms and accession numbers (locus tags). Other sequences are shown by their accession numbers, labels, and strain names. Desaturase genes that have been functionally characterized are indicated on the tree by their labels. Bootstrap values from neighbor-joining analyses are listed to the left of each node, with values more than 50 are shown.

**Figure 5:**
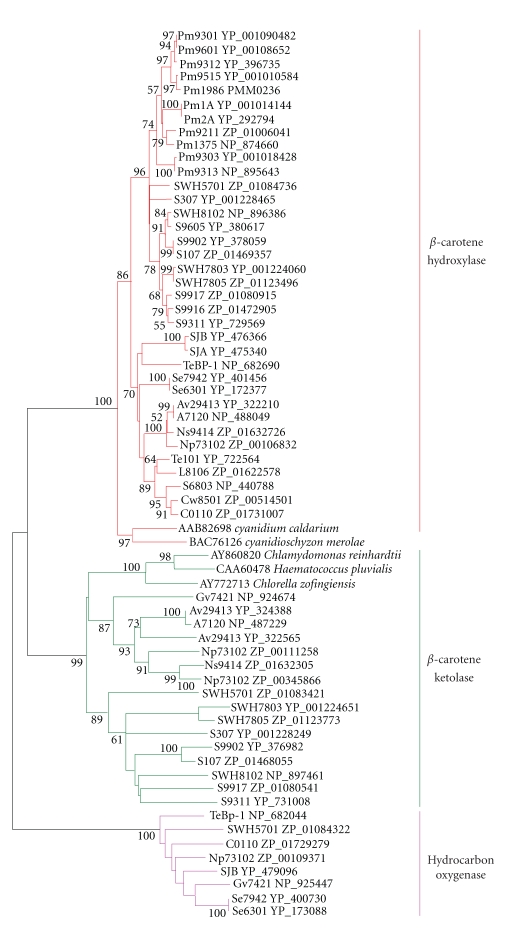
Minimum-evolution tree of *β*-carotene ketolase, *β*-carotene hydroxylase, and hydrocarbon oxygenase homologs of cyanobacteria and eukaryotic algae. About 220 positions spanning the three histidine-boxes were employed. Colored branches indicate different groups of proteins. Red: *β*-carotene hydroxylase, green: *β*-carotene ketolase, magenta: hydrocarbon oxygenase. Sequences from 37 sequenced cyanobacterial genomes are shown by their acronyms and accession numbers (locus tags). Other sequences are shown by their accession numbers, labels, and strain names. Desaturase genes that have been functionally characterized are indicated on the tree by their labels. Bootstrap values from minimum-evolution analyses are listed to the left of each node, with values more than 50 are shown.

**Figure 6:**
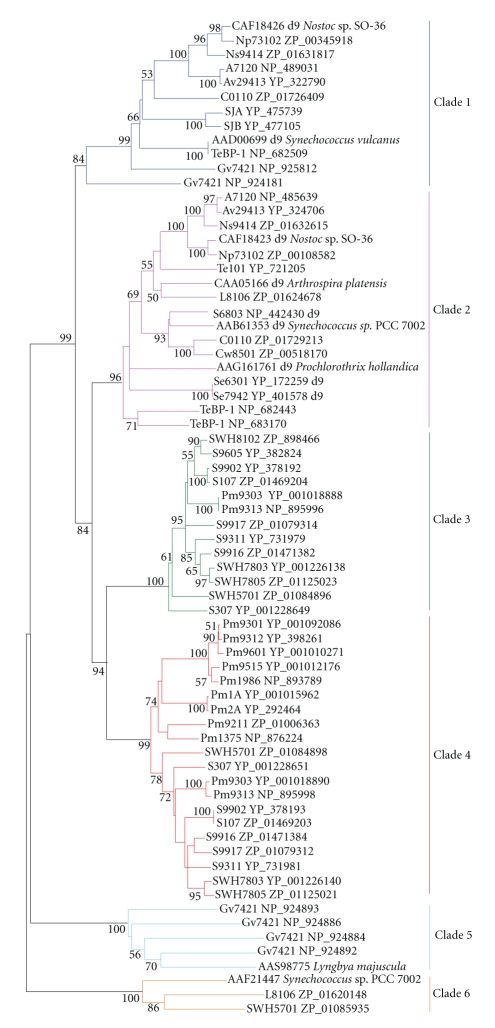
Neighbor-joining tree of Δ9-homologous genes of cyanobacteria and eukaryotic algae. About 250 positions spanning the three histidine-boxes were employed. Colored branches indicate different groups of proteins. Dark blue: Clade 1, magenta: Clade 2, green: Clade 3, red: Clade 4, light blue: Clade 5, orange: Clade 6. Sequences from 37 sequenced cyanobacterial genomes are shown by their acronyms and accession numbers (locus tags). Other sequences are shown by their accession numbers, labels, and strain names. Desaturase genes that have been functionally characterized are indicated on the tree by their labels. Bootstrap values from neighbor-joining analyses are listed to the left of each node, with values more than 50 are shown.

**Figure 7:**
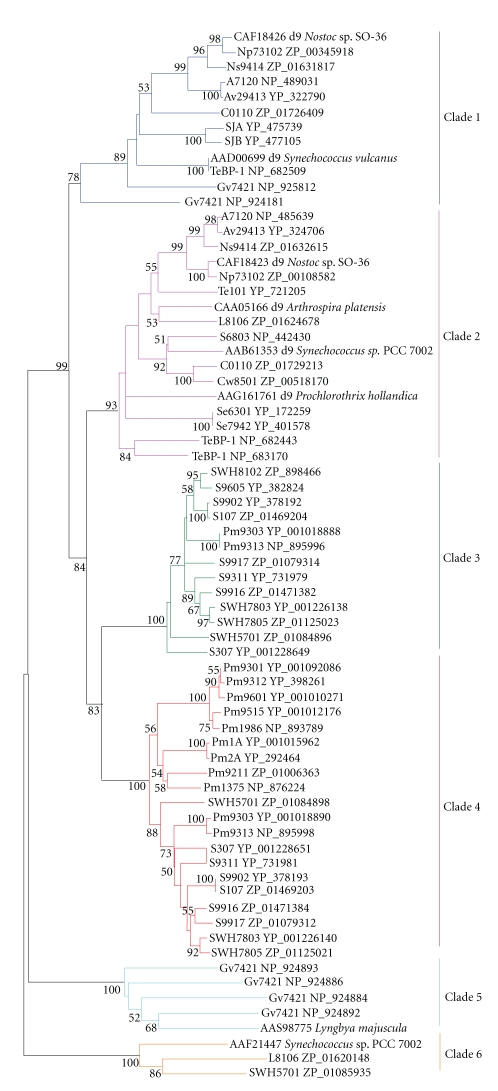
Minimum-evolution tree of Δ9-homologous genes of cyanobacteria and eukaryotic algae. About 250 positions spanning the three histidine-boxes were employed. Colored branches indicate different groups of proteins. Dark blue: Clade 1, magenta: Clade 2, green: Clade 3, red: Clade 4, light blue: Clade 5, orange: Clade 6. Sequences from 37 sequenced cyanobacterial genomes are shown by their acronyms and accession numbers (locus tags). Other sequences are shown by their accession numbers, labels, and strain names. Desaturase genes that have been functionally characterized are indicated on the tree by their labels. Bootstrap values from minimum-evolution analyses are listed to the left of each node, with values more than 50 are shown.

As shown in Figures 12 and 13, Δ9 desaturases clustered into a single-monophyletic group, thus were analyzed separately from other types of desaturases. Six clades could be identified within the Δ9 desaturase homologs from cyanobacteria based on high-bootstrap support values and a large degree of within-clade sequence identity (Figures 3, 6, and 7). Except for the genes from Clade 6 (ZP_01620148, ZP_01085935, and AAF21447) whose second residue of the second histidine-box was not arginine, the genes from other clades all matched the standard for Δ9 desaturase, that is, HR-X_3_-H, HR-X-HH, and HN-X-HH. Thus, genes from Clade 6 are assigned as hypothetical proteins with functions unknown.

The first clade was composed by one Δ9-homologous gene from eight N_2_-fixing cyanobacterial species (such as *Nostoc* sp. strain SO-36 and *Anabaena* sp. PCC 7120), *Thermosynechococcus elongatus* BP-1, *Synechococcus vulcanus*, and two genes from *Gloeobacter violaceus*. The amino acid identity of these genes ranged from 50% to 98% among various cyanobacterial species. It has been proven by previous research that the Δ9 desaturase gene from *Nostoc* sp. strain SO-36 in this clade introduced double bonds into fatty acids that are bound to the *sn*-2 position of the glycerol moiety of membrane glycerolipids [19]. Moreover, the three histidine-boxes of the gene from *Nostoc* sp. SO-36 were consistent with those of genes in Clade 1. Therefore, the genes of Clade 1 are presumed to act on fatty acids esterified to the *sn*-2 position of glycerolipids.

In Clade 2, one Δ9-homologous gene from *Prochlorothrix hollandica*, *Synechococcus* sp. PCC 7942, and *Synechococcus* sp. PCC 6301 clustered together with two genes from *Thermosynechococcus elongatus*, apart from the subgroup comprised of genes from nine N_2_-fixing cyanobacterial species (such as *Anabaena variabilis* and *Trichodesmium erythraeum*), *Synechocystis* sp. PCC 6803, *Synechococcus* sp. PCC 7002, and *Arthrospira platensis*. It has been demonstrated that *Thermosynechococcus elongatus* has three Δ9-homologous genes that consist of one c-type and two unspecified types. By contrast, * Synechococcus* sp. PCC 7942, *Synechococcus* sp. PCC 6301, and *Prochlorothrix hollandica* have only one Δ9-homologous gene, which is nonspecific with respect to *sn* positions, acting on fatty acids at both the *sn*-1 and *sn*-2 positions [19]. Δ9 homologs from another subgroup showed high similarity with amino acid identity from 53% to 98% among various cyanobacterial species. They are strongly homologous to the genes of *Synechocystis* sp. PCC 6803 (NP_442430), *Synechococcus* sp. PCC 7002 (AAB61353), and *Arthrospira platensis* (CAA05166) that encode Δ9 desaturases acting on C18 fatty acids at the *sn*-1 position. Moreover, the three histidine-boxes of these Δ9-homologous genes (HRX_3_HRSF, WXGXHRXHH, GEGWHNNHH) accorded with those inferred by Chintalapati et al. (2006) [19].

The Δ9-homologous genes from two unicellular marine cyanobacteria *Synechococcus* and *Prochlorococcus* constituted the third and fourth clades. Amino acid identity of genes from these two clades ranged from 54% to 98% and 65% to 99%, respectively. In addition, the two groups are closely related to Clade 2. Therefore, it is possible that these genes are homologous to the gene that encodes a Δ9 desaturase acting on C18 fatty acids at the *sn*-1 position or *sn*-1 and *sn*-2 positions of glycerolipids. In these two clades, 11 strains (nine *Synechococcus* and two low light-adapted *Prochlorococcus* strains) contained two Δ9-homologous genes, which clustered separately into two subgroups. It is possible that there are two paralogous genes of a common ancestor in some evolutionary lineages, such as *Synechococcus* sp. CC9605; however, one of them has been lost. Alternatively, acquirement of one gene from other organisms could have occurred in the evolutionary lineage, in which horizontal gene transfer (HGT) might have taken place.

Four genes of *Gloeobacter violaceus* PCC 7421 as well as JamB gene of *Lyngbya majuscula* integrated the fifth clade. JamB is a gene of jamaicamide biosynthetic gene cluster, and similar to a large family of membrane-associated desaturases that utilize a diiron active site to execute Δ5- or Δ9-fatty acid desaturation [20]. These genes fell into the group of proteobacterial stearoyl-CoA desaturases, far away from the other desaturase genes of cyanobacteria as analyzed by BLASTP program of NCBI (data not shown). It is probable that horizontal gene transfer (HGT) from other organisms like proteobacteria might have occurred.

Phylogenetic analyses from Figures 12 and 13 showed that Δ9 desaturases from cyanobacteria were grouped to those from green algae and higher plants, apart from red algae, diatoms, fungi, and animals. Among cyanobacterial Δ9 desaturases, the desaturase genes acting on fatty acids esterified to the *sn*-1 or *sn*-1 and *sn*-2 positions of glycerolipids (b-type or a-type) were placed in a basal position, while desaturase genes acting on fatty acids esterified to the *sn*-2 position of glycerolipids (c-type) were in the exoteric position, which indicates that a-type or b-type Δ9 desaturases may be ancestral to c-type desaturase.

### 3.3. Discovery of Candidate Genes for Δ12/*ω*3 Desaturases

**Figure 8:**
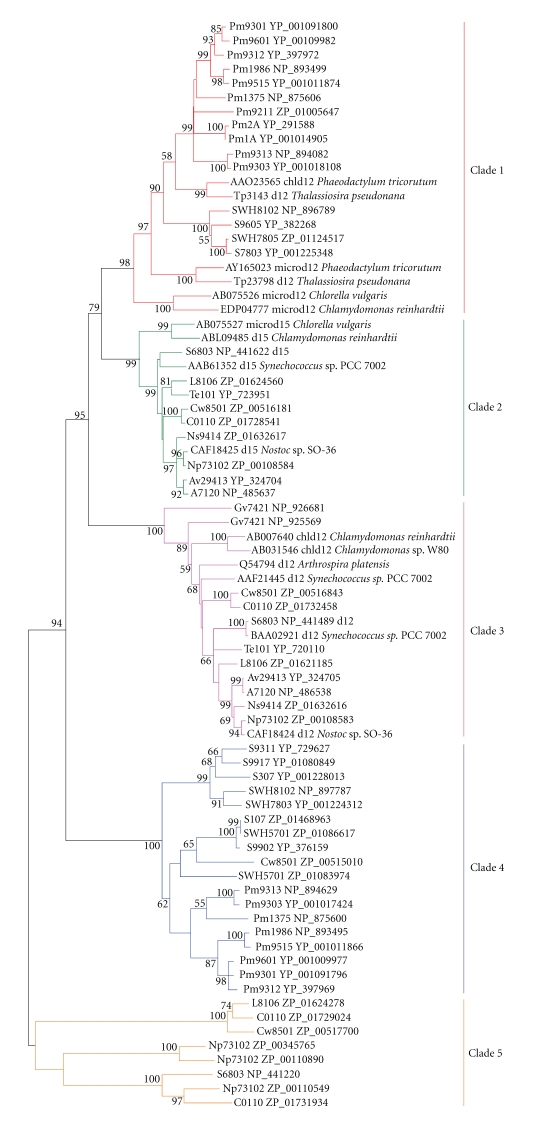
Neighbor-joining tree of Δ12 and Δ15 homologous genes of cyanobacteria and eukaryotic algae. About 300 positions spanning the three histidine-boxes were employed. Colored branches indicate different groups of proteins. Red: Clade 1, green: Clade 2, magenta: Clade 3, blue: Clade 4, orange: Clade 5. Sequences from 37 sequenced cyanobacterial genomes are shown by their acronyms and accession numbers (locus tags). Other sequences are shown by their accession numbers, labels, and strain names. Desaturase genes that have been functionally characterized are indicated on the tree by their labels. Bootstrap values from neighbor-joining analyses are listed to the left of each node, with values more than 50 are shown.

**Figure 9:**
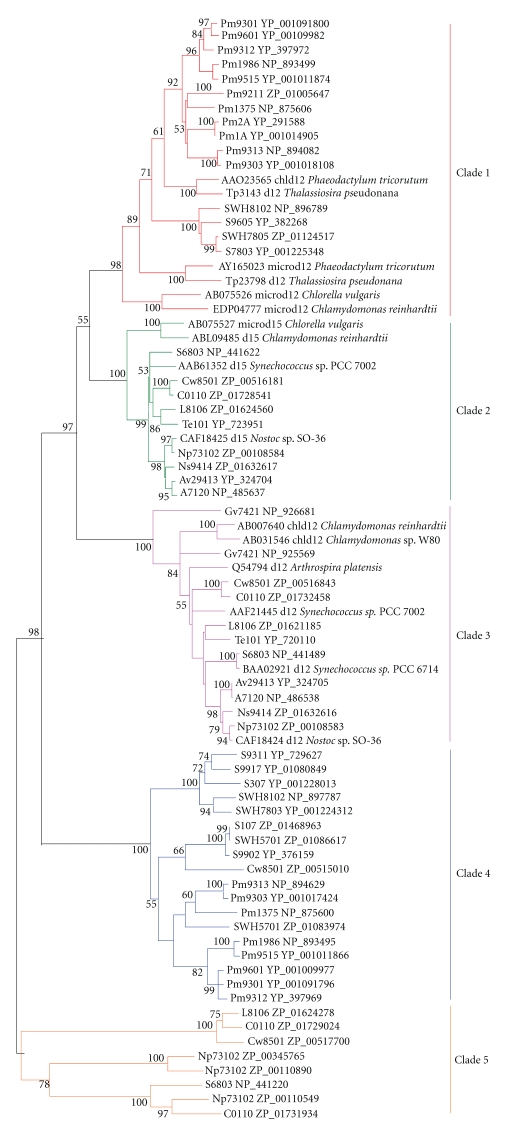
Minimum-evolution tree of Δ12 and Δ15 homologous genes of cyanobacteria and eukaryotic algae. About 300 positions spanning the three histidine-boxes were employed. Colored branches indicate different groups of proteins. Red: Clade 1, green: Clade 2, magenta: Clade 3, blue: Clade 4, orange: Clade 5. Sequences from 37 sequenced cyanobacterial genomes are shown by their acronyms and accession numbers (locus tags). Other sequences are shown by their accession numbers, labels, and strain names. Desaturase genes that have been functionally characterized are indicated on the tree by their labels. Bootstrap values from minimum-evolution analyses are listed to the left of each node, with values more than 50 are shown.

Observation on the phylogenetic tree of different membrane desaturases showed that Δ12 desaturases and Δ15 desaturases fell into the same clade (Figures 12 and 13), thus were analyzed together. As could be seen in Figures 8 and 9, the Δ12/*ω*3 desaturase homologs from cyanobacteria were classified into five different clades.

It was surprising that the first clade was constituted by the Δ12 homologs of marine cyanobacteria *Synechococcus*, *Prochlorococcus,* and the microsomal Δ12 desaturases of eukaryotic algae. Moreover, three histidine-boxes of the genes from cyanobacteria were represented as AHECGH, WX_2_SHX_2_HHX_3_N, and HX_2_HH (Figure 2 and Table 3), which were similar to those of microsome-type desaturases. Two partial amino acid sequences homologous to microsome-type Δ12 desaturases were revealed in *Prochlorococcus* *marinus* MIT 9211 (ZP_01005647 and ZP_01005648). One encoded an N-terminus region and the other encoded a C-terminus region. They may represent a single gene inferred from their close chromosome location of the graft genome, thus were designated as a unique gene with the accession number ZP_01005647.

The microsomal Δ12 desaturases are members of a large class of membrane-bound enzymes that contain a tripartite histidine sequence motif and two putative membrane-spanning domains. This group of membrane-bound enzymes includes desaturases, hydroxylases, epoxygenases, acetylenases, methyl oxidases and ketolases found in animals, fungi, plants, and bacteria [21–23]. The diverse reactions that these enzymes catalyze probably use a common reactive center [24]. Histidine-rich motifs are thought to form a part of the diiron center, where oxygen activation and substrate oxidation occur [25].

**Figure 10:**
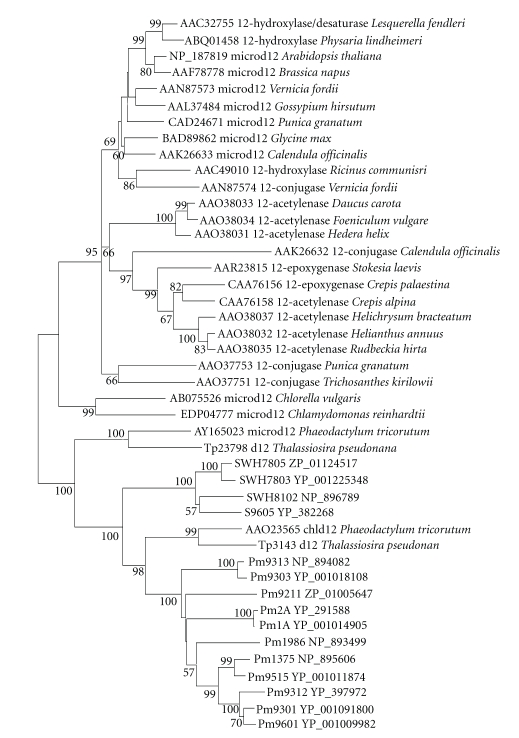
Neighbor-joining tree of Δ12 homologous genes of cyanobacteria, eukaryotic algae, and higher plants. About 300 positions spanning the three histidine-boxes were employed. Sequences from 37 sequenced cyanobacterial genomes are shown by their acronyms and accession numbers (locus tags). Other sequences are shown by their accession numbers, labels, and strain names. Desaturase genes that have been functionally characterized are indicated on the tree by their labels. Bootstrap values from neighbor-joining analyses are listed to the left of each node, with values more than 50 are shown.

**Figure 11:**
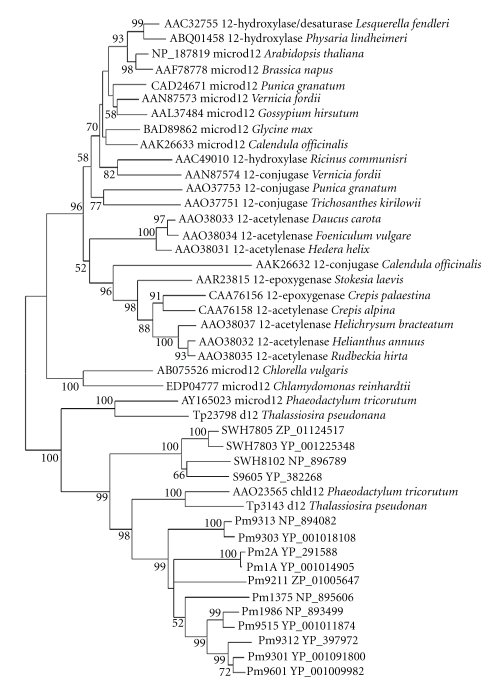
Minimum-evolution tree of Δ12 homologous genes of cyanobacteria, eukaryotic algae, and higher plants. About 300 positions spanning the three histidine-boxes were employed. Sequences from 37 sequenced cyanobacterial genomes are shown by their acronyms and accession numbers (locus tags). Other sequences are shown by their accession numbers, labels, and strain names. Desaturase genes that have been functionally characterized are indicated on the tree by their labels. Bootstrap values from minimum-evolution analyses are listed to the left of each node, with values more than 50 are shown.

To further clarify the role of genes in Clade 1, anotherphylogenetic tree was constructed by neighbor-joining (NJ) and minimum-evolution (ME) methods (Figures 10 and 11). It could be seen evidently from Figures 10 and 11 that the microsomal Δ12 desaturases from higher plants and some eukaryotic algae (such as green algae, *chlorella,* and *chlamydomonas*) fell into one group with Δ12 fatty acid hydroxylase, epoxygenase, acetylenase, and conjugase, while the genes of marine cyanobacteria clustered only with diatom plastidial and microsomal Δ12 desaturases [26]. Therefore, the microsomal Δ12 desaturases of some eukaryotic algae (such as diatom) might originate from cyanobacterial orthologs in Clade 1, and possibly horizontal gene transfer might have occurred from eukaryotic algae to *Synechococcus* and *Prochlorococcus* strains.

The *ω*3-homologous genes of cyanobacteria and eukaryotic algae constituted the second clade. Moreover, three histidine-boxes of the genes from cyanobacteria (FVVGHDCGHXSFS, HGWRISHRTHHXNTGN, and IHHXIGTHVAHHIF) established the standard for prokaryotic Δ15 desaturase (Figure 2 and Table 3). The third clade was integrated by the Δ12 homologs of cyanobacteria and the chloroplastic Δ12 desaturases of eukaryotic algae. Moreover, three histidine-boxes of these genes were consistent with those of plastidial Δ12 desaturase that were represented as HDCGH, HX_2_HH, and HXPHH.

**Figure 12:**
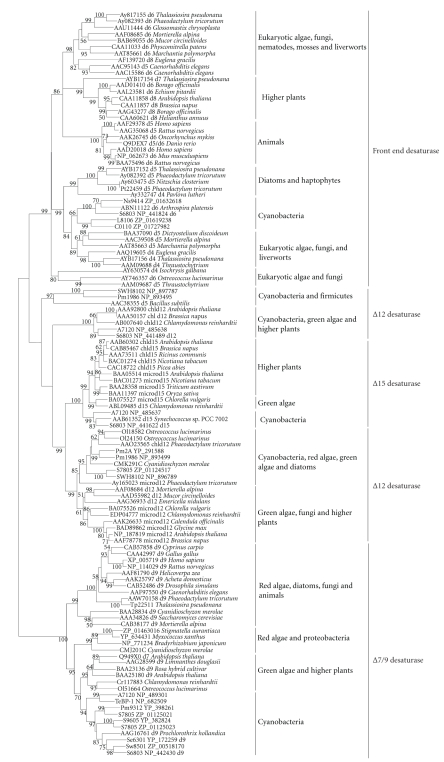
Neighbor-joining tree of membrane desaturases. About 330 positions spanning the three histidine-boxes were employed. Sequences from 37 sequenced cyanobacterial genomes are shown by their acronyms and accession numbers (locus tags). Other sequences are shown by their accession numbers, labels, and strain names. Desaturase genes that have been functionally characterized are indicated on the tree by their labels. Bootstrap values from neighbor-joining analyses are listed to the left of each node, with values more than 50 are shown.

**Figure 13:**
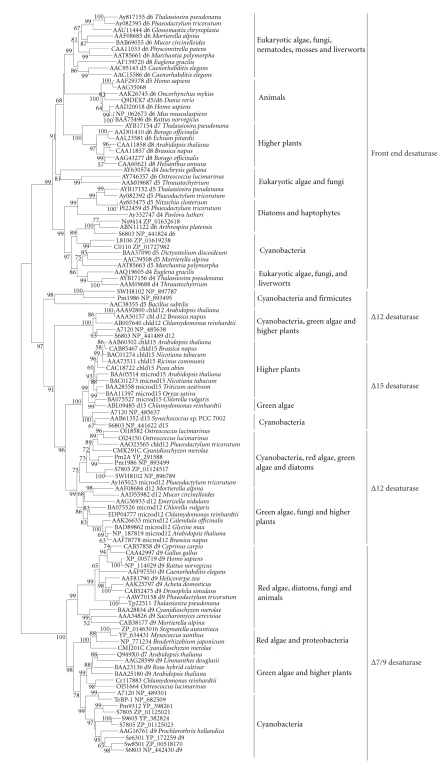
Minimum-evolution tree of membrane desaturases. About 330 positions spanning the three histidine-boxes were employed. Sequences from 37 sequenced cyanobacterial genomes are shown by their acronyms and accession numbers (locus tags). Other sequences are shown by their accession numbers, labels, and strain names. Desaturase genes that have been functionally characterized are indicated on the tree by their labels. Bootstrap values from minimum-evolution analyses are listed to the left of each node, with values more than 50 are shown.

The homologous genes from Clade 4 also had three histidine-motifs (FSLMHDCGHXSLF, WSX_2_HAXHHX_2_NG, and HX_2_HHLXERIPNYXL) (Figure 2 and Table 3) that were similar to those of the Δ12 desaturase. As shown in Figures 12 and 13, the genes of this clade clustered with *Bacillus subtilis* Δ5 desaturase. Aguilar et al. (1998) demonstrated that *Bacillus subtilis* possessed a single desaturase. Expression of the gene in *Escherichia coli* resulted in desaturation of palmitic acid moieties of the membrane phospholipids to give the novel mono-UFA cis-5-hexadecenoic acid, indicating that the gene product was a Δ5 acyl-lipid desaturase [27]. However, it is well known from freshwater cyanobacteria that only four distinct desaturases, Δ9, Δ12, Δ15, and Δ6, exist in cyanobacterial cells. Therefore, the relatively close phylogenetic relationship between genes of Clade 4 and Δ5 desaturase gene of *Bacillus subtilis* may be due to horizontal gene transfer and the function of these genes would require further work to fully characterize.

Three genes from *Nostoc punctiforme* ATCC 29133, two genes from *Cyanothece* sp. CCY0110, and one gene from *Synechocystis* sp. PCC 6803, *Crocosphaera watsonii* WH 8501, *Lyngbya* sp. PCC 8106 constituted the fifth clade. It has been proven by experiments that there is only one Δ12 desaturase in *Synechocystis* sp. PCC 6803 [13]. Additionally, the three histidine-motifs of these genes were HXXXH, HXXXHH, HXXHH, among which the amounts of residues between histidines from the second histidine-box were three, while that of known cyanobacterial Δ12 desaturase were two (HXXXH, HXXHH, HXXHH). Therefore, in our analysis they are assigned as hypothetical proteins and their functions need to be further investigated.

As indicated by Figures 12 and 13, the Δ12/*ω*3 desaturase subfamily was integrated by two main groups. Group 1 included the Δ12 desaturases from *Synechococcus*, *Prochlorococcus* and Δ5 desaturase from *Bacillus subtilis*. In Group 2, the Δ12 desaturases of cyanobacteria and the chloroplastic Δ12 desaturases of green algae, higher plants were in the basal position, leading to Cluster 1. In Cluster 2, the microsomal Δ12 desaturases of fungi, green algae, and higher plants set apart from Δ12 desaturases of *Synechococcus*, *Prochlorococcus*, *Cyanidioschyzon merolae*, *Ostreococcus*, *Thalassiosira pseudonana,* and *Phaeodactylum tricorutum*. Cluster 3 included the *ω*3 desaturases of cyanobacteria at the basal position, *ω*3 desaturases of green algae and both microsomal and chloroplastic *ω*3 desaturases of higher plants. Thus, the plastidial Δ12 desaturases are ancestral to the *ω*3 and microsomal Δ12 desaturases, and the *ω*3 desaturase of higher plants and green algae arose by independent gene duplication events from prokaryotic *ω*3 desaturase [28].

### 3.4. Discovery of Candidate Genes for Δ6 Desaturases

The “front-end” desaturases (Δ4, Δ5, Δ6, and Δ8 desaturases) formed a separate clade, and their phylogeny is complicated (Figures 12 and 13). It has been speculated that front-end desaturases may have the same origin, but their precise lineages are still unclear. There were just four prokaryotic Δ6 desaturases found from cyanobacterial genomes in our analysis: *Synechocystis* sp. PCC 6803 (NP_441824), *Cyanothece* sp. CCY0110 (ZP_01727982), *Lyngbya* sp. PCC 8106 (ZP_01619238), *Nodularia spumigena* CCY9414 (ZP_01632618), among which the function and molecular characteristics of Δ6 acyl-lipid desaturases from *Synechocystis* sp. PCC 6803 had been fully analyzed [13].

### 3.5. Occurrence and Phyletic Distribution of Fatty Acid Desaturases in Thirty Seven Cyanobacteria

**Figure 14:**
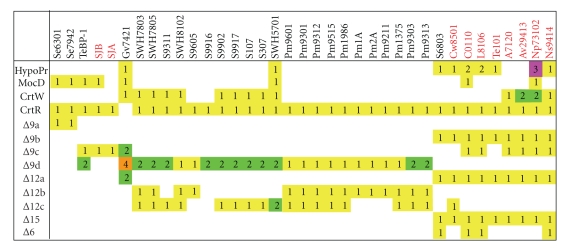
Diversity of different enzymes in thirty seven cyanobacteria. Distributions and amounts of different enzymes are marked by colors. One: red, two: green, three: magenta, four: orange. Names of nitrogen-fixing strains are marked in red. “HypoPr” represents hypothetical protein.

**Figure 15:**
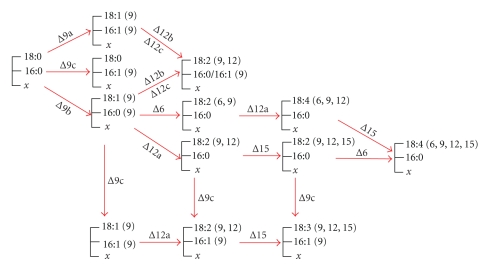
The acyl-lipid desaturation of fatty acids in cyanobacteria. Numbers around arrowhead indicate the positions at which a double bond is introduced. Δ9a : desaturation occurring on both the *sn*-1 and the *sn*-2 positions of glycerolipids, Δ9b: desaturation occurring on the *sn*-1 position of glycerolipids, Δ9c : desaturation occurring on the *sn*-2 position of glycerolipids, Δ9d: genes with desaturation *sn*-position of glycerolipids unspecified. Δ12a : Clade 3 of Δ12 homologous genes, Δ12b: Clade 1 of Δ12 homologous genes, Δ12c : Clade 4 of Δ12 homologous genes.

In this study, thirty one unicellular and six filamentous cyanobacterial genomes were searched by bioinformatic approach for the putative fatty acid desaturases involved in polyunsaturated fatty acid synthesis. 193 protein sequences were obtained from the 37 cyanobacterial genomes, 120 of which were annotated as fatty acid desaturase. The pathway of acyl-lipid desaturation and the distribution of desaturases among different cyanobacterial species were speculated and summarized in Figures 14 and 15. Among these cyanobacteria, the Δ9 desaturase existed in 37 species of cyanobacteria. The Δ12, Δ15 and Δ6 desaturases existed in 31, 9, and 4 species of cyanobacteria, respectively. Based on functional criteria and the position of the clade integrated by Δ9 desaturases, Δ9 desaturase is assumed to be the ancestor of the remaining desaturases [28]. The functions performed by the latter three desaturases could have been obtained in some organisms along the evolutionary lineages.

Twenty seven of the investigated cyanobacteria come from the marine environment. These are 11 unicellular *Prochlorococcus* strains, 11 unicellular marine *Synechococcus* strains, *Cyanothece* sp. CCY0110, *Crocosphaera watsonii* WH 8501, *Trichodesmium erythraeum* IMS101, *Lyngbya* sp. PCC 8106, and *Nodularia spumigena* CCY9414. The other strains are from freshwater, soil, rock, hot spring, or symbiont.

In the 16S rRNA tree, marine *Synechococcus* and *Prochlorococcus* make a monophyletic group supported by a comparatively high-statistical confidence value, 100% (Figure 1). The two genera are proposed to diverge from a common phycobilisome-containing ancestor. While marine *Synechococcus* still uses phycobilisomes as light-harvesting antennae, members of the *Prochlorococcus* genus lack phycobilisomes and use a different antenna complex that possesses derivatives of chlorophyll a and b. They are the dominant picophytoplankton in the world’s open oceans. Carbon fixation is dominated by them and together they have been shown to contribute between 32 and 80% of the primary production in oligotrophic oceans [29–32]. *Synechococcus* are distributed ubiquitously throughout oceanic regions, ranging from polar through temperate to tropical waters and are generally more abundant in nutrient-rich surface waters than oligotrophic areas, whilst *Prochlorococcus* are largely confined to a 40°N∼40°S latitudinal band, being generally absent from brackish or well-mixed waters. *Prochlorococcus* also generally extend deeper in the water column than *Synechococcus* [33, 34].

*Prochlorococcus* have been divided into two genetically and physiologically distinct groups: high- and low-B/A ecotypes, which were originally named for their difference in optimal growth irradiance (low- and high-light adapted, resp.) [35, 36]. High-B/A isolates, with larger ratios of chl *b*/*a*_2_, are able to grow at extremely low irradiances (less than 10 umol of quanta [Q] m^−2^  s^−1^) and preferentially thrive at the bottom of the euphotic zone (80–200 m) at dimmer light but in a nutrient-rich environment [37, 38]. Low-B/A isolates, have lower chl *b*/*a*_2_ ratios, are able to grow maximally at higher light intensities, and occupy the upper, well illuminated but nutrient-poor 100-m layer of the water column [37, 38]. In the 16S rRNA tree, high-light-adapted *Prochlorococcus* sp. arises from a low-light-adapted clade (Figure 1). *Prochlorococcus marinus* strains AS9601, MIT 9312, MIT 9301, MIT 9515, and CCMP1986 belong to low-B/A ecotype. Their genome sizes vary from 1.6 Mb to 1.7 Mb, smaller than that of the low light-adapted strains (1.7 Mb to 2.6 Mb). They all contain two types of desaturases, one Δ9 desaturases and two Δ12 desaturases (b-type and c-type). Strains NATL1A, NATL2A, MIT 9211, CCMP1375, MIT 9303, and MIT 9313 belong to high-B/A ecotype. Only b-type Δ12 desaturase exists in strain NATL1A, NATL2A, and MIT 9211; while two Δ9 desaturases exist in strain MIT 9303 and MIT 9313, which have larger genome size (2.6 Mb and 2.4 Mb) compared to other high-B/A ecotypes.

The marine *Synechococcus* isolates have themselves been classified into three groups, designated marine cluster -A, -B, and -C (MC-A, MC-B, MC-C), based on the composition of the major light harvesting pigments, an ability to perform a novel swimming motility, whether they have an elevated salt requirement for growth, and G+C content [39]. The marine cluster A group (mol% G+C = 55–62), phycoerythrin-containing strains, has an elevated salt (Na^+^, Cl^−^, Mg^2+^ and Ca^2+^) requirement for growth and occur abundantly within the euphotic zone of both open-ocean and coastal waters [40–44]. This cluster is additionally diverse in that ratios of phycourobilin to phycoerythrobilin chromophores differ among phycoerythrins of different strains [45, 46]. The marine cluster B (mol% G+C = 63–69.5) includes halotolerant strains that possess phycocyanin but lack phycoerythrin and appear confined to coastal waters. A further cluster, marine cluster C (MC-C) has been distinguished by its low % G+C (47.5–49.5) containing strains from brackish or coastal marine waters [39]. These latter environments have been relatively poorly studied so far and are likely underrepresented in cultured *Synechococcus* isolates [33]. The b-type Δ12 desaturase only exists in strains WH 7803, WH 7805, WH 8102, and CC9605. Except for strains RS9916 and CC9605, other strains all contain c-type Δ12 desaturase, two copies of which exist in strain WH 5701 (MC-B) whose genome (30 Mb) is larger than other *Synechococcus* strains (22 Mb–26 Mb). The unique characteristics can be observed in strain RS9916 that contains only Δ9 fatty acid desaturase.

The pathway of acyl-lipid desaturation for marine cyanobacteria *Prochlorococcus* and *Synechococcus* differs obviously from that of other cyanobacteria, indicating the different phylogenetic histories of the two genera from other cyanobacteria. At present, few fatty acid composition of these unicellular cyanobacteria has been determined yet, as functionally characterized genes. Therefore, the analysis on fatty acids in these cyanobacteria should provide more meaningful information for further research.

The two closely related freshwater *Synechococcus elongatus* strains PCC 6301 and PCC 7942 branch outside the marine picophytoplankton group (Figure 1), which suggests that marine cyanobacteria may diverge from the freshwater cyanobacterial ancestor. The gene arrangement and nucleotide sequence of *Synechococcus elongatus* PCC 6301 are nearly identical to those of *Synechococcus elongatus* PCC 7942, except for the presence of a 188.6 kb inversion. Genome-wide screening only recognizes one a-type Δ9 desaturase in these two strains.

Three thermophilic unicellular strains, *Thermosynechococcus elongatus* BP-1 and two *Synechococcus* Yellowstone species, are most closely related to *Gloeobacter violaceus* sp. PCC 7421, and phylogenetically distinct from other cyanobacterial lineages (Figure 1). They were all isolated from the hot spring. Additionally, the latter two thermophilic strains are capable of N_2_ fixation with a diurnal rhythm. Genes for three types of fatty acid desaturases (*desA*, *desB*, and *desD*) are missing in contrast with mesophilic *Synechocystis*, although the fourth type (*desC*) is found in *Synechococcus* and *Thermosynechococcus elongtus*. This agrees with the absence of highly unsaturated fatty acids in lipids, which are popular in many thermophiles [47]. *Synechococcus* sp. JA-2-3B′a(2-13) as well as JA-3-3Ab contains one c-type Δ9 desaturase, whereas *Thermosynechococcus elongtus* contains three copies, one c-type and two unspecified types. At lower temperatures, cyanobacteria desaturate the fatty acids of membrane lipids to compensate for the decrease in membrane fluidity [48]. While at higher temperatures, the membrane fluidity increased, it is unnecessary to desaturate the fatty acids of membrane lipids to produce more unsaturated fatty acids. So the thermophilic strains lack highly unsaturated fatty acids in lipids and contain only one Δ9 desaturase in contrast with mesophilic strains, which probably due to their thermic habitats.

*Gloeobacter violaceus* sp. PCC 7421 was originally isolated from calcareous rock in Switzerland [49, 50]. It is an unusual unicellular cyanobacterium for the absence of thylakoid membranes, and its phycobilisomes and photosystem reaction centers are localized in the plasma membrane [51, 52]. It is also remarkable that Sulfoquinovosyl diacylglycerol (SQDG), which is thought to have an important role in photosystem stabilization, is absent in *Gloeobacter* while the content of polyunsaturated fatty acids (PUFA) is high [53]. The data of the fatty acid composition of *Gloeobacter violaceus* are few in number and contradictory. In one case, linoleic and *α*-linolenic acids were found [53]. In other work, linoleic and *γ*-linolenic acids were identified [54]. The occurrence of *α*-linolenic or *γ*-linolenic acid confirms that there must be a gene in the strain that is functionally similar to the *ω*3 desaturase or Δ6 desaturase. Two types of desaturases, six Δ9 desaturases (two c-types and four unspecified types) and two Δ12 desaturases (a-type), were recognized from this strain. One hypothetical protein (NP_923117) was also found, but the three histidine-motifs of it (HDAGH, HNQLHH, HTAHH) did not agree with the standards for a front-end or *ω*3 desaturase. It is this protein or another protein that performs the same function as the front-end or *ω*3 desaturase, which need further investigation. The types and amounts of desaturases in *Gloeobacter violaceus* sp. PCC 7421 are distinct to those of other cyanobacteria (Figure 14). This result may accord with the conclusion that this organism is one of the earliest ones that diverged from the cyanobacterial line [55].

Nine of the 37 cyanobacteria studied here are known to fix nitrogen (Figure 1). Four Nostocales, *Nostoc punctiforme* ATCC 29133, *Anabaena* sp. PCC 7120, *Anabaena variabilis* ATCC 29413, and *Nodularia spumigena* CCY9414, are heterocyst-forming filamentous diazotroph; the other five are nonheterocystous nitrogen fixers, which are filamentous strains *Trichodesmium erythraeum* IMS101, *Lyngbya* sp. PCC 8106, unicellular strains *Crocosphaera watsonii* WH 8501, *Cyanothece* sp. CCY0110 along with thermophic *Synechococcus* strains JA-2-3B′a(2-13) and JA-3-3Ab.

The diazotrophic filamentous cyanobacteria, which can form terminally differentiated, nondividing heterocysts in response to nitrogen deprivation and the ensuing intracellular accumulation of 2-oxoglutarate [56], have almost the largest genome sizes (53 Mb–90 Mb) and are isolated from soil (*Anabaena* PCC7120), from fresh water (*Anabaena variabilis* ATCC 29413), from a plant-cyanobacterial symbionsis (*Nostoc punctiforme* PCC73102), or from the surface of Baltic sea (*Nodularia spumigena* CCY9414). Three types of desaturases (Δ9, Δ12, and Δ15) exist in *Anabaena* sp. PCC 7120, *Anabaena variabilis* ATCC 29413, and *Nostoc punctiforme* ATCC 29133, with the exception that *Nodularia spumigena* CCY9414 contains four types of desaturases (Δ9, Δ12, Δ15, and Δ6). Moreover, phylogenetic analysis shows that the desaturase genes of the same type all cluster together for these four strains, indicating a recent common ancestor for *Anabaena* and *Nostoc* [57].

*Trichodesmium erythraeum* IMS101 and *Lyngbya* sp. PCC 8106, which belong to the Oscillatoriales, both fix N_2_ and do not form heterocysts (Figure 1). *Trichodesmium*, but not *Lyngbya*, is known to fix nitrogen in differentiated cells called diazocytes. Like heterocysts, diazocytes are the exclusive carriers of nitrogenase and fix nitrogen aerobically in the light, and show morphological and physiological changes [58].

Unicellular strains *Crocosphaera watsonii* WH 8501, *Cyanothece* sp. CCY0110, and *Synechocystis* sp. PCC 6803 belong to the Chroococcaces (Figure 1), among which the former two strains fix nitrogen presumably at night while growing photosynthetically during the day. Three types of desaturases (Δ9, Δ12, and Δ15) exist in *Crocosphaera watsonii* WH 8501 and *Trichodesmium erythraeum*, while four types of desaturases (Δ9, Δ12, Δ15, and Δ6) exist in *Lyngbya* sp. PCC 8106, *Cyanothece* sp. CCY0110 and *Synechocystis* sp. PCC 6803. It is worth noting that the c-type Δ12 desaturase is identified exclusively in *Crocosphaera watsonii* WH 8501, which may be due to horizontal gene transfer (HGT) from marine cyanobacteria *Prochlorococcus* and *Synechococcus*.

In conclusion, the filamentous or N_2_-fixing cyanobacteria usually possess more types of fatty acid desaturases than unicellular species. The main role of fatty acid desaturase of cyanobacteria is to modulate the fluidity of membranes, which helps to improve tolerance to physiological stressors such as low temperature, high light-induced photoinhibition, salt-induced damage, or desiccation. Thus, the amounts and types of fatty acid desaturases are various among different cyanobacterial species. This evolution scheme might have formed under the force adapting to distinct environments.

## Figures and Tables

**Table 1:** Lists of putative desaturase genes from thirty seven cyanobacterial genomes.

Species	Locus tag	Accession	DNA coordinates	Length	Proposed function
*Anabaena* sp. PCC 7120	all4991	NP_489031	5963080⋯5963937	857	d9
	all1599	NP_485639	1879629⋯1880447	818	d9
	all1598	NP_485638	1878346⋯1879398	1052	d12
	all1597	NP_485637	1876897⋯1877976	1079	d15
	alr3189	NP_487229	3858986⋯3859762	776	crtW
	alr4009	NP_488049	4829483⋯4830322	839	crtR

*Anabaena variabilis* ATCC 29413	Ava_2277	YP_322790	2832413⋯2833270	857	d9
	Ava_4212	YP_324706	5282348⋯5283166	818	d9
	Ava_4211	YP_324705	5281066⋯5282118	1052	d12
	Ava_4210	YP_324704	5279614⋯5280693	1079	d15
	Ava_2048	YP_322565	2535646⋯2536410	764	crtW
	Ava_3888	YP_324388	4842189⋯4842965	776	crtW
	Ava_1693	YP_322210	2121129⋯2122049	920	crtR

*Crocosphaera watsonii* WH 8501	CwatDRAFT_1377	ZP_00518170	3068⋯3892	824	d9
	CwatDRAFT_3226	ZP_00516843	22017⋯23066	1049	d12
	CwatDRAFT_5150	ZP_00515010	150888⋯151982	1049	d12
	CwatDRAFT_3625	ZP_00516181	10760⋯11809	1049	d15
	CwatDRAFT_1857	ZP_00517700	1398⋯2231	834	hypothetical protein
	CwatDRAFT_5424	ZP_00514501	315629⋯316522	893	crtR

*Gloeobacter violaceus* strain PCC 7421	gvip390	NP_925812	3057506⋯3058357	851	d9
	gvip170	NP_924181	1312274⋯1313095	822	d9
	gll1946	NP_924892	2071551⋯2072504	953	d9
	gll1947	NP_924893	2072509⋯2073507	998	d9
	gll1938	NP_924884	2060880⋯2061839	959	d9
	gll1940	NP_924886	2063884⋯2064876	992	d9
	gvip364	NP_925569	2779580⋯2780638	1058	d12
	gvip506	NP_926681	3944843⋯3945910	1058	d12
	gll0171	NP_923117	161268⋯162440	1173	hypothetical protein
	gll2501	NP_925447	2660474⋯2661475	1001	mocD
	gvip239	NP_924674	1833712⋯1834485	773	crtW

*Nostoc punctiforme* ATCC 29133(PCC 73102)	Npun02000467	ZP_00345918	175651⋯176532	881	d9
	Npun02005010	ZP_00108582	41108⋯41929	821	d9
	Npun02005011	ZP_00108583	42265⋯43326	1061	d12
	Npun02005012	ZP_00108584	43524⋯44603	1080	d15
	Npun02001904	ZP_00345765	63255⋯64310	1056	hypothetical protein
	Npun02001905	ZP_00110890	64537⋯65574	1038	hypothetical protein
	Npun02002344	ZP_00110549	77763⋯78863	1101	hypothetical protein
	Npun02003462	ZP_00109371	76020⋯76964	945	mocD
	Npun02000865	ZP_00345866	139810⋯140571	762	crtW
	Npun02001326	ZP_00111258	55604⋯56392	788	crtW
	Npun02006805	ZP_00106832	23657⋯24556	899	crtR

*Prochlorococcus marinus* str. NATL1A	NATL1_21421	YP_001015962	1799954⋯1800733	780	d9
	NATL1_10821	YP_001014905	992775⋯993992	1218	d12
	NATL1_03151	YP_001014144	291853⋯292884	1032	crtR

*Prochlorococcus marinus* strain NATL2A	PMN2A_1271	YP_292464	1227545⋯1228474	929	d9
	PMN2A_0393	YP_291588	388657⋯389874	1217	d12
	PMN2A_1603	YP_292794	1566557⋯1567588	1031	crtR

*Prochlorococcus* marinus MIT 9211	P9211_09157	ZP_01006363	1417821⋯1418765	944	d9
	P9211_05577	ZP_01005647	779723⋯780334	611	d12
	P9211_05582	ZP_01005648	780304⋯780729	425	d12
	P9211_07547	ZP_01006041	1108444⋯1109469	1015	crtR

*Prochlorococcus marinus* str. MIT 9301	P9301_18621	YP_001092086	1588713⋯1589651	939	d9
	P9301_15761	YP_001091800	1328773⋯1329939	1167	d12
	P9301_15721	YP_001091796	1326076⋯1327182	1107	d12
	P9301_02581	YP_001090482	239249⋯239974	726	crtR

*Prochlorococcus marinus* str. MIT 9303	P9303_28951	YP_001018890	2560285⋯2561250	966	d9
	P9303_28931	YP_001018888	2558615⋯2559535	921	d9
	P9303_14121	YP_001017424	1208715⋯1209800	1086	d12
	P9303_21081	YP_001018108	1869188⋯1870330	1143	d12
	P9303_24321	YP_001018428	2137288⋯2138328	1041	crtR

*Prochlorococcus marinus* str. MIT 9312	PMT9312_1764	YP_398261	1656076⋯1657014	938	d9
	PMT9312_1476	YP_397972	1385670⋯1386845	1175	d12
	PMT9312_1473	YP_397969	1382796⋯1383902	1106	d12
	PMT9312_0238	YP_396735	229042⋯229842	800	crtR

*Prochlorococcus marinus* str. MIT 9313	PMT2172	NP_895996	2299082⋯2300002	920	d9
	PMT2174	NP_895998	2300938⋯2301717	779	d9
	PMT0249	NP_894082	278544⋯279683	1139	d12
	PMT0797	NP_894629	872385⋯873470	1085	d12
	PMT1816	NP_895643	1920323⋯1921363	1040	crtR

*Prochlorococcus marinus* str. AS9601	A9601_18811	YP_001010271	1616719⋯1617657	939	d9
	A9601_15921	YP_001009982	1355480⋯1356514	1035	d12
	A9601_15871	YP_001009977	1352826⋯1353932	1107	d12
	A9601_02571	YP_001008652	238284⋯239117	834	crtR

*Prochlorococcus marinus* str. MIT 9515	P9515_18621	YP_001012176	1650943⋯1651929	987	d9
	P9515_15601	YP_001011874	1376566⋯1377693	1128	d12
	P9515_15521	YP_001011866	1371646⋯1372752	1107	d12
	P9515_02681	YP_001010584	247534⋯248433	900	crtR

*Prochlorococcus marinus* subsp. *marinus* str. CCMP1375 (SS120)	Pro1833	NP_876224	1690865⋯1691797	932	d9
	Pro1208	NP_875600	1116904⋯1118016	1112	d12
	Pro1214	NP_875606	1121144⋯1122250	1106	d12
	Pro0266	NP_874660	261189⋯262223	1034	crtR

*Prochlorococcus marinus* subsp. *marinus* str. CCMP1986 (MED4)	PMM1672	NP_893789	1604745⋯1605731	986	d9
	PMM1382	NP_893499	1331162⋯1332340	1178	d12
	PMM1378	NP_893495	1325388⋯1326494	1106	d12
	PMM0236	/	228281⋯229270	989	crtR

*Synechococcus elongatus* strain PCC 7942	Synpcc7942_2561	YP_401578	2639146⋯2639982	836	d9
	Synpcc7942_1713	YP_400730	1781317⋯1782219	902	mocD
	Synpcc7942_2439	YP_401456	2514276⋯2515271	995	crtR

*Synechococcus elongatus* strain PCC 6301	syc1549_d	YP_172259	1676804⋯1677640	837	d9
	Syc2378_c	YP_173088	2534831⋯2535691	861	mocD
	syc1667_c	YP_172377	1801757⋯1802752	996	crtR

*Synechococcus* sp. BL107	BL107_07284	ZP_01469203	490784⋯491566	782	d9
	BL107_07289	ZP_01469204	491936⋯492721	785	d9
	BL107_06084	ZP_01468963	247334⋯248356	1022	d12
	BL107_14110	ZP_01468055	331111⋯331884	773	crtW
	BL107_08054	ZP_01469357	636707⋯637738	1031	crtR

*Synechococcus* sp. CC9311	sync_2793	YP_731981	2458778⋯2459710	932	d9
	sync_2791	YP_731979	2457075⋯2457986	911	d9
	sync_0336	YP_729569	344430⋯345449	1019	crtR
	sync_0396	YP_729627	408306⋯409505	1199	d12
	sync_1804	YP_731008	1621108⋯1621869	761	crtW

*Synechococcus* sp. CC9605	Syncc9605_2541	YP_382824	2358792⋯2359703	911	d9
	Syncc9605_1972	YP_382268	1793076⋯1794221	1145	d12
	Syncc9605_0286	YP_380617	292821⋯293870	1049	crtR

*Synechococcus* sp. CC9902	Syncc9902_2191	YP_378192	2099771⋯2100673	902	d9
	Syncc9902_2192	YP_378193	2100902⋯2101825	923	d9
	Syncc9902_0141	YP_376159	149723⋯150724	1001	d12
	Syncc9902_0972	YP_376982	954015⋯954788	773	crtW
	Syncc9902_2058	YP_378059	1964618⋯1965730	1112	crtR

*Synechococcus* sp. JA-2-3B′a(2-13)	CYB_0861	YP_477105	894187⋯895071	884	d9
	CYB_2914	YP_479096	3011594⋯3012520	926	mocD
	CYB_0102	YP_476366	118335⋯119306	971	crtR

*Synechococcus* sp. JA-3-3Ab	CYA_2349	YP_475739	2357019⋯2357912	893	d9
	CYA_1931	YP_475340	1944066⋯1945040	974	crtR

*Synechococcus* sp. RCC307	SynRCC307_2395	YP_001228651	2091372⋯2092274	903	d9
	SynRCC307_2393	YP_001228649	2089667⋯2090581	915	d9
	SynRCC307_1757	YP_001228013	1538507⋯1539562	1056	d12
	SynRCC307_1993	YP_001228249	1729342⋯1730103	762	crtW
	SynRCC307_2209	YP_001228465	1915148⋯1916167	1020	crtR

*Synechococcus* sp. RS9916	RS9916_36767	ZP_01471384	1050409⋯1051341	932	d9
	RS9916_36757	ZP_01471382	1048603⋯1049568	965	d9
	RS9916_39311	ZP_01472905	116650⋯117675	1025	crtR

*Synechococcus* sp. RS9917	RS9917_06370	ZP_01079314	447782⋯448705	923	d9
	RS9917_06360	ZP_01079312	446060⋯446992	932	d9
	RS9917_03333	ZP_01080849	99968⋯101047	1079	d12
	RS9917_00687	ZP_01080541	64826⋯65563	737	crtW
	RS9917_03663	ZP_01080915	166940⋯167902	962	crtR

*Synechococcus* sp. WH 5701	WH5701_02025	ZP_01084898	299319⋯300257	787	d9
	WH5701_02015	ZP_01084896	297579⋯298532	953	d9
	WH5701_14646	ZP_01083974	104382⋯105539	1157	d12
	WH5701_16535	ZP_01086617	164⋯1186	1022	d12
	WH5701_06521	ZP_01085935	65353⋯66231	878	hypothetical protein
	WH5701_02369	ZP_01084322	42300⋯43271	971	mocD
	WH5701_04005	ZP_01083421	43734⋯44519	785	crtW
	WH5701_01215	ZP_01084736	138584⋯139615	1031	crtR

*Synechococcus* sp. WH 7803	SynWH7803_2417	YP_001226140	2249293⋯2250087	795	d9
	SynWH7803_2415	YP_001226138	2247475⋯2248386	912	d9
	SynWH7803_0589	YP_001224312	594539⋯595603	1065	d12
	SynWH7803_1625	YP_001225348	1496144⋯1497139	996	d15
	SynWH7803_0928	YP_001224651	871421⋯872167	747	crtW
	SynWH7803_0337	YP_001224060	361336⋯362337	1002	crtR

*Synechococcus* sp. WH 7805	WH7805_10184	ZP_01125021	209067⋯209999	932	d9
	WH7805_10194	ZP_01125023	210769⋯211680	911	d9
	WH7805_06186	ZP_01124768	405535⋯406059	524	d12
	WH7805_04931	ZP_01124517	184338⋯185516	1178	d12
	WH7805_01197	ZP_01123773	3991⋯4734	743	crtW
	WH7805_07481	ZP_01123496	193165⋯194193	1028	crtR

*Synechococcus* sp. WH 8102	SYNW2377	NP_898466	2286168⋯ 2287028	860	d9
	SYNW0696	NP_896789	679330⋯680478	1148	d12
	SYNW1696	NP_897787	1631011⋯1632147	1136	d12
	SYNW1368	NP_897461	1354793⋯1355527	734	crtW
	SYNW0291	NP_896386	291323⋯292354	1031	crtR

*Synechocystis* sp. PCC 6803	sll0541	NP_442430	2822579⋯2823535	956	d9
	slr1350	NP_441489	1746308⋯1747363	1055	d12
	sll1441	NP_441622	1895520⋯1896599	1079	d15
	sll0262	NP_441824	2120067⋯2121146	1079	d6
	Sll1611	NP_441220	1462136⋯1463245	1110	hypothetical protein
	sll1468	NP_440788	981691⋯982629	938	crtR

*Thermosynechococcus elongatus* strain BP-1	tll1719	NP_682509	1800682⋯1801521	839	d9
	tlr2380	NP_683170	2490209⋯2491048	839	d9
	tlr1653	NP_682443	1733919⋯1734767	848	d9
	tlr1254	NP_682044	1300388⋯1301308	920	mocD
	tlr1900	NP_682690	1986642⋯1987529	887	crtR

*Trichodesmium erythraeum* IMS101	Tery_1437	YP_721205	2173203⋯2174015	812	d9
	Tery_0142	YP_720110	207806⋯208861	1055	d12
	Tery_4492	YP_723951	6931402⋯6932475	1073	d15
	Tery_3898	YP_723406	6024293⋯6025342	1050	hypothetical protein
	Tery_2925	YP_722564	4543239⋯4544114	875	crtR

*Lyngbya* sp. PCC 8106	L8106_03152	ZP_01624678	2253⋯3071	818	d9
	L8106_27002	ZP_01621185	94912⋯95955	1043	d12
	L8106_10697	ZP_01624560	6961⋯8043	1082	d15
	L8106_14825	ZP_01619238	100018⋯101133	1115	d6
	L8106_06180	ZP_01620148	172993⋯173604	611	hypothetical protein
	L8106_18641	ZP_01624278	13290⋯14111	821	hypothetical protein
	L8106_30215	ZP_01622578	23391⋯24185	794	crtR

*Nodularia spumigena* CCY9414	N9414_19077	ZP_01631817	16235⋯17026	791	d9
	N9414_07494	ZP_01632615	317⋯1135	818	d9
	N9414_07499	ZP_01632616	1303⋯2427	1124	d12
	N9414_07504	ZP_01632617	2618⋯3688	1070	d15
	N9414_07509	ZP_01632618	4087⋯5178	1091	d6
	N9414_18293	ZP_01629726	29633⋯30223	590	hypothetical protein
	N9414_07726	ZP_01632305	4851⋯5633	782	crtW
	N9414_01572	ZP_01632726	697⋯1587	890	crtR

*Cyanothece* sp. CCY0110	CY0110_10577	ZP_01726409	185891⋯186724	834	d9
	CY0110_05582	ZP_01729213	74180⋯75004	825	d9
	CY0110_10917	ZP_01732458	7951⋯9000	1050	d12
	CY0110_00445	ZP_01728541	90142⋯91191	1050	d15
	CY0110_24056	ZP_01727982	158769⋯159887	1119	d6
	CY0110_13441	ZP_01729024	60390⋯61220	831	hypothetical protein
	CY0110_27283	ZP_01731934	15787⋯16914	1128	hypothetical protein
	CY0110_11357	ZP_01729279	9512⋯10513	1002	mocD
	CY0110_08481	ZP_01731007	25752⋯26747	996	crtR

**Table 2:** List of organisms (except the above thirty seven cyanobacteria) and protein sequences analyzed in this study. Note: micro represents Microsomal, chl represents Chloroplastic, “uncertain” means that the function of the gene is uncertain.

Species	Accession no/locus tag	Label	Accession no/locus tag	Label
*Arabidopsis thaliana*	BAA25180	d9	AAB60302	chld15
	Q949X0	d7	BAA05514	microd15
	AAA92800	chld12	CAA11858	d8
	NP_187819	microd12		

*Thalassiosira pseudonana*	Tp22511	d9	AY817152	d5
	Tp23798	d12	AY817155	d6
	Tp3143	d12	AY817154	d8
	AY817156	d4		

*Phaeodactylum tricorutum*	AAW70158	d9	AY082393	d6
	AAO23565	chld12	AY082392	d5
	AY165023	microd12	Pt22459	d5

*Chlamydomonas reinhardtii*	Cr117883	uncertain	ABL09485	d15
	AB007640	chld12	AY860820	crtW
	EDP04777	microd12		

*Synechococcus* sp. PCC 7002	AAB61353	d9	AAF21445	d12
	AAF21447	uncertain	AAB61352	d15

*Nostoc* sp. SO-36	CAF18426	d9	CAF18425	d15
	CAF18423	d9	CAF18424	d12

*Mortierella alpina*	CAB38177	d9	AAF08684	d12
	AAF08685	d6	AAC39508	d5

*Cyanidioschyzon merolae*	BAA28834	d9	CMK291C	d12
	CMJ201C	d9	BAC76126	crtR

*Arthrospira platensis*	CAA05166	d9	Q54794	d12
	ABN11122	d6		

*Ostreococcus* *lucimarinus*	Ol51664	uncertain	Ol24150	d12
	Ol18582	d12		

*Caenorhabditis elegans*	AAF97550	d9	AAC15586	d6
	AAC95143	d5		

*Rattus norvegicus*	NP_114029	d9	BAA75496	d6
	AAG35068	d5		

*Homo sapiens*	XP_005719	d9	AAD20018	d6
	AAF29378	d5		

*Brassica napus*	AAA50157	chl d12	AAF78778	microd12
	CAA11857	d8		

*Chlorella vulgaris*	AB075526	microd12	AB075527	microd15
*Chlamydomonas* sp. W80	AB031546	chld12		
*Synechocystis* sp. PCC 6714	BAA02921	d12		
*Mucor circinelloides*	AAD55982	d12	BAB69055	d6
*Emericella nidulans*	AAG36933	d12		
*Glycine max*	BAD89862	microd12		
*Calendula officinalis*	AAK26633	microd12		
*Gossypium hirsutum*	AAL37484	microd12		
*Nicotiana tabacum*	BAC01274	chld15	BAC01273	microd15
*Brassica juncea*	CAB85467	chld15		
*Picea abies*	CAC18722	chld15		
*Ricinus communis*	AAA73511	chld15	AAC49010	12-hydroxylase
*Triticum aestivum*	BAA28358	microd15		
*Oryza sativa*	BAA11397	microd15		
*Vernicia fordii*	AAN87573	microd12	AAN87574	12-conjugase
*Punica granatum*	CAD24671	microd12	AAO37753	12-conjugase
*Lesquerella fendleri*	AAC32755	12-hydroxylase/desaturase		
*Physaria lindheimeri*	ABQ01458	12-hydroxylase		
*Crepis palaestina*	CAA76156	12-epoxygenase		
*Stokesia laevis*	AAR23815	12-epoxygenase		
*Daucus carota*	AAO38033	12-acetylenase		
*Foeniculum vulgare*	AAO38034	12-acetylenase		
*Hedera helix*	AAO38031	12-acetylenase		
*Helianthus annuus*	AAO38032	12-acetylenase	CAA60621	d8
*Helichrysum bracteatum*	AAO38037	12-acetylenase		
*Rudbeckia hirta*	AAO38035	12-acetylenase		
*Crepis alpina*	CAA76158	12-acetylenase		
*Calendula officinalis*	AAK26632	12-conjugase		
*Trichosanthes kirilowii*	AAO37751	12-conjugase		
*Acheta domesticus*	AAK25797	d9		
*Cyprinus carpio*	CAB57858	d9		
*Drosophila simulans*	CAB52475	d9		
*Gallus gallus*	CAA42997	d9		
*Helicoverpa zea*	AAF81790	d9		
*Rosa hybrid cultivar*	BAA23136	d9		
*Saccharomyces cerevisiae*	AAA34826	d9		
*Limnanthes douglasii*	AAG28599	d9		
*Prochlorothrix hollandica*	AAG16761	d9		
*Lyngbya majuscula*	AAS98775	d9		
*Synechococcus vulcanus*	AAD00699	d9		
*Thraustochytrium* sp. ATCC21685	AAM09688	d4	AAM09687	d5
*Euglena gracilis*	AAQ19605	d4	AF139720	d8
*Pavlova lutheri*	AY332747	d4		
*Isochrysis galbana* strain CCMP1323	AY630574	d4		
*Marchantia polymorpha*	AAT85663	d5	AAT85661	d6
*Nitzschia closterium* f. minutissima	AY603475	d5		
*Dictyostelium discoideum*	BAA37090	d5		
*Bacillus subtilis*	AAC38355	d5		
*Danio rerio*	Q9DEX7	d5/d6		
*Borago officinalis*	AAD01410	d6	AAG43277	d8
*Oncorhynchus mykiss*	AAK26745	d6		
*Mus musculus*	NP_062673	d6		
*Glossomastix chrysoplasta*	AAU11444	d6		
*Ostreococcus tauri*	AY746357	d6		
*Physcomitrella patens*	CAA11033	d6		
*Echium pitardii*	AAL23581	d6		
*Chlorella zofingiensis*	AY772713	crtW		
*Cyanidium caldarium*	AAB82698	crtR		
*Haematococcus pluvialis*	CAA60478	crtW		
*Myxococcus xanthus* DK 1622	YP_634431	uncertain		
*Stigmatella aurantiaca* DW4/3-1	ZP_01463016	uncertain		
*Bradyrhizobium japonicum* USDA 110	NP_771234	uncertain		

**Table 3:** Conserved motifs of membrane desaturases in cyanobacteria. Note: X represents an unspecified amino acid. Δ9-1: clade 1 of Δ9 homologous genes, Δ9-2: clade 2 of Δ9 homologous genes, Δ9-3: clade 3 of Δ9 homologous genes, Δ9-4: clade 4 of Δ9 homologous genes, Δ9-5: clade 5 of Δ9 homologous genes, Δ12a : clade 3 of Δ12 homologous genes, Δ12b: clade 1 of Δ12 homologous genes, Δ12c : clade 4 of Δ12 homologous genes, Δ15: Δ15 desaturase, Δ6: Δ6 desaturase.

Name	H-box1	H-box2	H-box3
*β*-carotene ketolase	TGLFIX_2_HDXMH	K(N)HX_2_HH	CY(F)H(N)FGYHXEHH
*β*-carotene hydroxylase	GTVIHDAS(C)HX_2_AH	RVHL(M)Q(E)HHXHVN	GQNYHLI(V)HHLWPSI(V)PW
hydrocarbon oxygenase	HECXHRTAFA	FY(F)RRYHXWHHRXT	MWNMPF(Y)HXEHHL(F)
Δ9-1	GICLGYHRLLXHKSF	WX_3_HRXHHAX_3_D	YGEGWHNNHHX_2_PX_5_GX_2_WWE
Δ9-2	GXTLGXHRX_3_HRSF	WXGXHRXHHX_2_SD	GEGWHNNHHX_4_SARHGXXWWE
Δ9-3	TVLGVTLGLHRLXAHRS	WX_2_LHRHHHX_2_SDQ	WVAXLSFGEGWHNNHHAXPXSARHGL
Δ9-4	CLGVTXGYHRLLXHRX_2_	WXGLHRHHHXFSDT	WVAALTFGEGWHNNHHAXPXSA
Δ9-5	GX_4_GXHRXFXHX_2_F	WX_3_HRXHHX_3_D	GESWHNNHHXFX_3_AX_2_G
Δ12a	FVXGHDCGHRSF	WRX_2_HX_2_HHX_2_TN	HXPHHX_4_IPXYNLR
Δ12b	WVXAHECGHXAFH	WX_2_SHX_2_HHX_3_N	HX_2_HHX_4_PHYXA
Δ12c	FSLMHDCGHXSLF	WSX_2_HAXHHX_2_NG	HX_2_HHLXERIPNYXL
Δ15	FWXLFVVGHDCGHXSFS	HGWRISHRTHHXNTGN	IHHXIGTHVAHHIF
Δ6	HDX_2_HX_3_S	WX_3_HX_2_LHHXYTNI	GGLNXQ(H)X_2_HHLFPXICH
